# Chlorogenic Acid’s Role in Metabolic Health: Mechanisms and Therapeutic Potential

**DOI:** 10.3390/nu17203303

**Published:** 2025-10-21

**Authors:** Katarzyna Zalewska, Maciej Kulawik, Julia Gierszewska, Zofia Gramala, Oliwia Kalus, Michał Karpiński, Joanna Maćkowiak, Antoni Staniewski, Zofia Szymańska, Barbara Zalewska, Wei Lu, Judyta Cielecka-Piontek, Przemysław Zalewski

**Affiliations:** 1 Department of Pharmacognosy and Biomaterials, Poznan University of Medical Sciences, Rokietnicka 3, 60-806 Poznan, Poland; 2 Doctoral School, Poznan University of Medical Sciences, Bukowska 70 St., 60-812 Poznan, Poland; 3 Department of Food Science and Engineering, School of Agriculture and Biology, Shanghai Jiao Tong University, Shanghai 200240, China

**Keywords:** chlorogenic acid, polyphenols, metabolic syndrome, antioxidant activity, obesity, diabetes

## Abstract

Chlorogenic acid (CGA), an ester of caffeic and quinic acids, is a dietary polyphenol abundant in coffee, tea, fruits, vegetables, and medicinal plants, with 5-O-caffeoylquinic acid (5-CQA) as its predominant isomer. This review aims to summarize current knowledge on the biological activities, mechanisms of action, and potential therapeutic applications of CGA in the prevention and management of metabolic disorders. CGA demonstrates a broad spectrum of biological activities relevant to human health. Its mechanisms of action involve modulation of oxidative stress and key cellular signaling pathways, as well as regulation of metabolic processes, contributing to improved insulin sensitivity, lipid balance, and overall energy homeostasis. These properties make CGA a promising agent against metabolic syndrome (type 2 diabetes, obesity, hypertension, and dyslipidemia) which is a major global health challenge. Despite its health benefits, CGA’s oral bioavailability remains limited, prompting research into optimized extraction methods, novel formulations, and structural modifications. Current evidence supports its safety even at high doses, reinforcing its potential as a nutraceutical, functional food ingredient, and adjunctive therapeutic compound in chronic disease management.

## 1. Introduction

**Figure 1:**
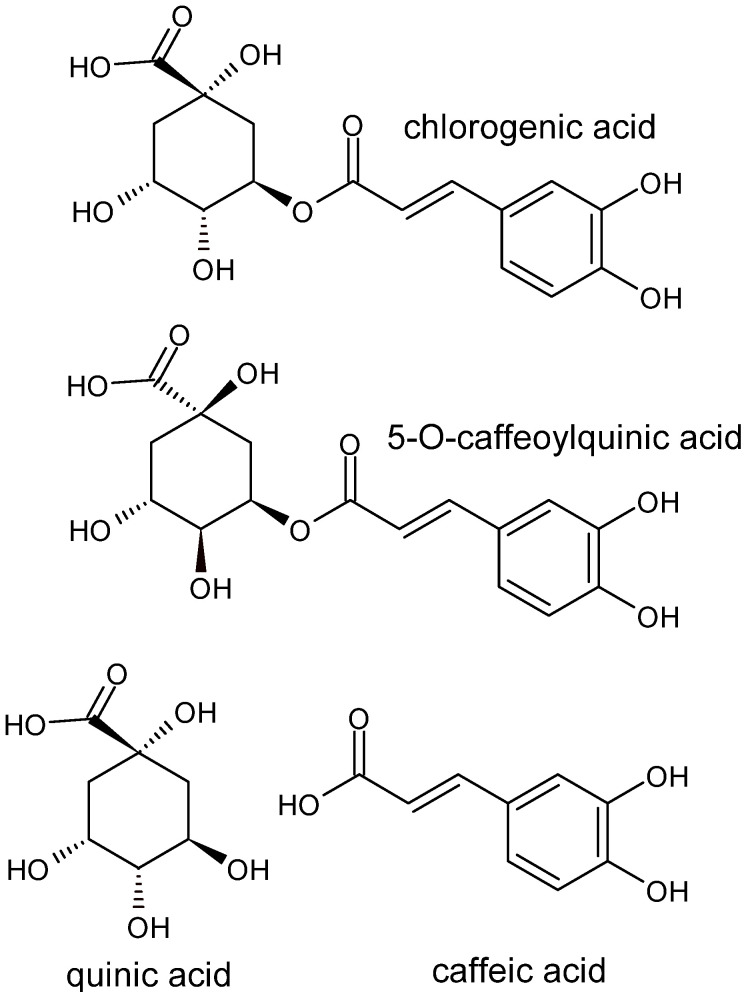
Chemical Structures of Chlorogenic Acid (CGA), 5-O-Caffeoylquinic Acid (5-CQA), Quinic Acid and Caffeic Acid (CA). CGA is an ester formed through the linkage of the carboxyl group of caffeic acid with the hydroxyl group at the C-5 position of quinic acid, highlighting its dual phenolic and acidic nature responsible for biological activity.

Chlorogenic acid (CGA), an ester of caffeic acid (CA) and quinic acid (see Figure 1), belongs to the group of polyphenols commonly found in the human diet, especially in coffee, tea, fruits, vegetables, and numerous medicinal plants [1]. It occurs in various isomers, among which 5-O-caffeoylquinic acid (5-CQA) is the most abundant form in plant-based foods and beverages [2]. CGA is considered one of the most important dietary phenolic compounds, exhibiting a wide range of biological activities, such as antioxidant, anti-inflammatory, anticancer, antibacterial, hepatoprotective, cardioprotective and neuroprotective effects, and modulation of lipid and glucose metabolism [3,4,5].

Studies indicate that CGA may play a significant role in the prevention and treatment of many chronic diseases, including metabolic syndrome (type 2 diabetes, obesity, hypertension, and dyslipidemia), cardiovascular diseases [6,7,8]. The mechanisms of CGA action include inhibition of oxidative stress, regulation of inflammatory responses through modulation of the NF-κB pathway and activation of the Nrf2 pathway, as well as effects on cellular energy metabolism through regulation of AMP-activated protein kinase (AMPK) activity [9,10,11]. Moreover, CGA may support metabolic homeostasis, improve insulin sensitivity, limit fat deposition in tissues, and influence the lipid profile [12,13].

Despite its numerous health benefits, the oral bioavailability of CGA remains relatively low, mainly due to its binding to plant cell wall components and microbiota-dependent metabolism [1,14,15,16]. Therefore, there is an intensive search for new extraction methods, formulations, and structural modifications to increase the stability and bioavailability of this compound, fully using its therapeutic potential [17].

In recent years, there has been growing interest in CGA in both basic and clinical research, owing to its promising pharmacological properties and safety [4,8,18]. It is used as a component of dietary supplements, nutraceuticals, and functional food additives, as well as a potential adjunct in therapies for chronic diseases and cancers [19,20].

Considering the global burden of metabolic disorders, the preventive and therapeutic potential of CGA is of particular relevance. Metabolic conditions including obesity, type 2 diabetes, hypertension, and dyslipidemia continue to pose significant public health challenges. Their development is associated with chronic oxidative stress, inflammation, and dysregulation of energy metabolism—mechanisms influenced by CGA, highlighting the potential role of this polyphenol in the prevention and treatment of these conditions. Untreated metabolic syndrome significantly increases the risk of developing various cancers and cardiovascular diseases, as well as decreases the quality of patients’ lives [21].

Obesity is recognized as a global epidemic, affecting hundreds of millions of people, and by 2030, it may encompass up to one billion individuals. Its characteristic features, such as ectopic fat deposition and adipose tissue dysfunction, promote the development of cardiovascular diseases [22]. Currently, about 880 million adults worldwide live with obesity (BMI ≥ 30 kg/m^2^), representing 16% of the population, and affected individuals have an increased risk of atherosclerosis, hypertension, coronary artery disease, arrhythmia, and heart failure [23]. Excessive growth of white adipose tissue leads to its dysfunction, chronic inflammation, and insulin resistance, increasing the risk of type 2 diabetes, cardiovascular diseases, fatty liver, and cancers [24]. Over the last four decades, obesity prevalence in the USA has tripled, now affecting over 42% of the population, and is associated with chronic inflammation and metabolic disturbances [25]. Moreover, obesity affects cardiovascular risk through changes in metabolic status, and individuals with impaired metabolic fitness have a significantly higher risk of developing heart and vascular diseases [26].

Hypertension is one of the most common cardiovascular diseases, affecting over one billion adults worldwide and is associated with an increased risk of heart disease, stroke, and organ damage [27]. Despite its high prevalence, hypertension often remains undiagnosed, leading to severe complications and premature death [28]. In 2019, only 54% of patients had a diagnosis, 42% received treatment, and only 21% effectively controlled their blood pressure, what highlights significant gaps in care [29]. Resistant hypertension affects 10–15% of patients and is associated with higher cardiovascular risk and comorbidities, such as obesity and chronic kidney disease [30]. Furthermore, the low efficacy of therapy, the limitations of the mechanisms of available drugs, and the development of more personalized treatment strategies requires further research and improvements [31].

Type 2 diabetes (T2DM) is a chronic metabolic disorder mainly resulting from insulin resistance, caused by genetic predisposition, unhealthy diet, and lack of physical activity, leading to chronic hyperglycemia, oxidative stress, and vascular damage [32]. In 2021, the disease affected approximately 536.6 million adults worldwide, and by 2045, this number may rise to 783.2 million [33]. In recent years, a significant increase in incidence among individuals younger than 40 years has also been observed [34]. Chronic hyperglycemia leads to severe macro- and microangiopathic complications, such as coronary artery disease, stroke, retinopathy, nephropathy, neuropathy, and diabetic foot syndrome. Type 2 diabetes requires consideration of both insulin secretion disorders and its ineffective utilization, and antioxidants are increasingly recognized for their role in the management of diabetic complications [35].

Dyslipidemia—encompassing abnormalities in cholesterol and triglyceride levels, is another significant risk factor for cardiovascular diseases, including coronary artery disease and stroke [36]. Lipid disorders are common and important for cardiovascular risk assessment, and contemporary diagnostic approaches consider both genetic and secondary factors, as well as additional parameters such as apoB and lipoprotein(a) [37]. Hypercholesterolemia, defined as elevated LDL levels ≥ 130 mg/dL, is one of the main factors contributing to atherosclerosis and other cardiovascular complications [38]. Dyslipidemia, as part of metabolic syndrome, significantly contributes to global mortality due to heart and vascular diseases, which in some regions, such as India, has nearly doubled in recent decades [39].

While numerous studies have explored the general health effects of chlorogenic acid, few have comprehensively examined its specific role in the context of the metabolic syndrome. This review therefore focuses on the connection between CGA and metabolic syndrome–associated disorders, integrating current evidence from both preclinical and clinical research.

## 2. Chlorogenic Acid in the Prevention and Management of Obesity

Obesity and related metabolic disorders are multifactorial conditions involving complex interactions between diet, lifestyle, genetics, inflammation, gut microbiota composition, and energy metabolism. Chlorogenic acid, a naturally occurring polyphenol, has gained increasing attention for its potential role in modulating these processes. Research demonstrates that CGA may influence body weight regulation through multiple pathways, including modulation of gut microbiota, reduction of inflammation, regulation of adipogenesis, and stimulation of thermogenesis. Moreover, CGA’s interaction with other bioactive compounds may enhance its anti-obesity effects, offering promising synergistic strategies. Both preclinical and clinical studies support the beneficial impact of CGA on weight control, body composition, and metabolic health.

### 2.1. CGA Effect on Gut Microbiota

**Figure 2:**
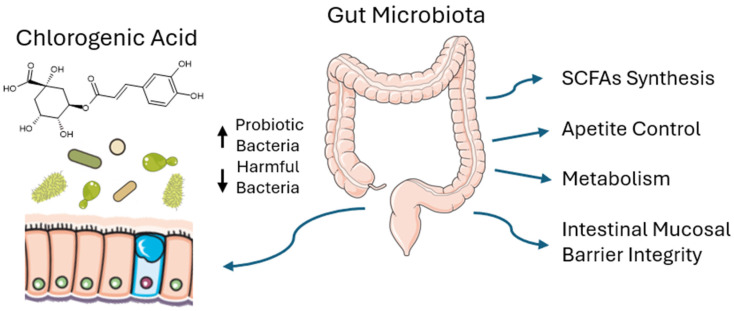
Mechanism of Chlorogenic Acid Action on Gut Microbiota and Its Anti-Obesity Effects. CGA modulates gut microbiota, increasing beneficial bacteria and reducing harmful strains, which boosts SCFA production, supports fat metabolism, strengthens the intestinal barrier, and reduces inflammation, contributing to anti-obesity effects and protection against metabolic disorders. Upward arrows (↑) indicate an increase in probiotic bacteria, while downward arrows (↓) indicate a decrease in harmful bacteria.

Research shows (see Figure 2) that gut microbiota and its dysregulation may be correlated with obesity—and thus with metabolic syndrome [11,40,41]. Although the exact mechanism remains unclear, studies also suggest a connection between gut microbiota and insulin sensitivity [42], inflammatory bowel disease [43], and colorectal cancer [44].

CGA affects the gut microbiota and, consequently, also obesity. According to in vivo studies in mice, CGA modifies the composition of the microbiota by, among other things, increasing the abundance of probiotic bacteria such as *Bifidobacterium* and *Lactobacillus*, while reducing the abundance of bacterial strains found in obese patients and animals, such as *Desulfovibrionaceae*, *Ruminococcaceae*, *Lachnospiraceae*, and *Erysipelotrichaceae* [11]. A study [45] revealed that Bifidobacterium sp. are able to grow in presence of CGA and have special enzymes that allow them to metabolize it. A later study [8] also demonstrated that CGA reduced the number of *Lachnospiraceae* (which are elevated in obesity [46]) and increased the number of *Muribaculaceae* and *Akkermansiaceae* (which have been negatively correlated with obesity, are anti-inflammatory, and improve the intestinal mucus barrier function) [47,48]. Another study showed that CGA reduced the amount of harmful bacteria such as *Escherichia coli* and, to a lesser extent, *Enterococcus* [49].

Furthermore, in the same study [8], CGA increased the levels of bacteria involved in the synthesis of short-chain fatty acids (SCFAs) (e.g., *Faecalibaculum*, *Dubosiella*). SCFAs support appetite control and energy metabolism through various mechanisms. SCFAs increase the secretion of the incretin hormone glucagon-like peptide (GLP)-1 [50], and inhibit the secretion of neuropeptide Y [51], which reduces appetite and counteracts obesity. Through GPR41/GPR43 receptors in adipose tissue, SCFAs increase leptin secretion [52], which is also associated with appetite suppression and enhanced fat burning. SCFAs have a beneficial effect on the integrity of the intestinal mucosal barrier, which protects against endotoxemia and inflammation—conditions linked to insulin resistance and obesity [53]. Propionate and butyrate (both SCFAs) activate intestinal gluconeogenesis, improving fat utilization [54], and butyrate additionally activates AMPK, inhibits HDAC, and increases PPARα expression, promoting fat oxidation [52].

Gut microbiota dysbiosis is also present in non-alcoholic fatty liver disease (NAFLD), which is closely linked to obesity [55]. Studies have shown that an increase in probiotic bacteria—such as *Bifidobacterium, Lactobacillus*—has a beneficial effect on NAFLD and NASH [56,57]. As previously mentioned, CGA increases the abundance of probiotic bacteria and also has a positive effect on the intestinal barrier integrity, protecting against endotoxemia. Both of these CGA effects could potentially protect against NAFLD, although the direct effect of CGA on NAFLD has not been fully investigated or confirmed.

### 2.2. Anti-Inflammatory Effect of CGA

One of the factors predisposing to obesity is a persistent inflammatory state [58]. Many plant-derived compounds exhibit relatively high antioxidant potential, which may reduce the risk of metabolic disorders [59]. CGA is a very effective agent in treating and preventing inflammation. It can inhibit nitric oxide (NO) synthesis in macrophages [60]. Chlorogenic acid contained in coffee may also exert an anti-obesity effect by reducing the production of reactive oxygen species (ROS) [61].

It has been shown that the main metabolites of chlorogenic acid formed in the large intestine, e.g., dihydrocaffeic acid, dihydroferulic acid, dihydroxyhippuric acid, have antioxidant properties and can reduce levels of pro-inflammatory cytokines such as IL-1β, IL-6, MCP-1. In an experiment conducted on the 3T3-L1 preadipocyte cell line under oxidative stress conditions—in this case using tumor necrosis factor α—it was shown that chlorogenic acid metabolites can reduce the production of ROS, and increase glutathione and glutathione peroxidase levels [62]. A 2023 study demonstrated that chlorogenic acid can also reduce the activity of zinc-dependent metalloproteinases (MMPs)—their excessive activity can be observed in diseases characterized by inflammation. One of the key mechanisms is the reduction in ROS levels, which can inhibit the expression of MMP genes as well as directly bind polyphenols to them and block their enzymatic activity. The authors highlight the potential future use of polyphenols as anti-inflammatory drugs [63].

### 2.3. CGA Effects on Metabolism, Adipogenesis and Thermogenesis

It has been demonstrated that in HepG2 hepatocytes, CGA increases energy metabolism by enhancing the activity of mitochondrial enzymes such as citrate synthase, isocitrate dehydrogenase, and malate dehydrogenase [64]. The same study showed that exposure to CGA led to an increase in proteins involved in the glycolytic cycle and mitochondrial ATP production, indicating an acceleration of metabolism. Another study also showed that CGA effectively inhibits triglyceride accumulation in HepG2 cells [65], and in adipocytes, it was observed that both caffeic acid (CA) and CGA increase the number and activity of mitochondria [66]. Thus, research indicates that CGA leads to faster metabolism and greater energy expenditure, resulting in reduced fat storage.

Chlorogenic acid may also exert anti-obesity effects through its influence on adipogenesis. This occurs via the mechanism of reducing the conversion of precursors into mature adipocytes [67]. In a 2019 study, it was demonstrated that chlorogenic acid and gallic acid most strongly inhibited preadipocyte differentiation among all the tested chemical compounds [63]. CGA inhibits adipogenesis mainly by suppressing the production of adipogenesis-related pathways such as PPAR-γ, adipocyte protein 2, fatty acid synthase, and lipoprotein lipase [68,69]. Inhibition of adipogenesis may also occur through reduction in oxidative stress by CGA, leading to decreased ROS levels and lower activity of transcription factors involved in adipogenesis [70]. Chlorogenic acid contained in an extract from *I. britannica* flowers may also inhibit adipogenesis by modulating its early stages, particularly mitotic clonal expansion [71].

CGA increases the level of phosphorylated (active) AMPK [66], which correlates with enhanced lipolysis and decreased lipid accumulation in adipocytes. AMPK inhibits the key enzyme of lipogenesis—acetyl-CoA carboxylase (ACC)—reducing fatty acid synthesis, while increasing the activity of lipolytic enzymes—ATGL and HSL—and the expression of genes involved in thermogenesis—UCP-1 and PGC-1α [72].

It has also been shown that CGA promotes the browning of adipose tissue, which has a beneficial impact on obesity [73]. Brown adipose tissue (BAT) and beige adipose tissue (BeAT) are capable of burning energy and participate in non-shivering thermogenesis [74]. Upon exposure to CA and CGA, the expression of markers typical of BAT—UCP1, PGC-1α, PPARα, CIDEA, and CD137—increased, while the expression of PPARγ (which promotes white adipose tissue development) and other factors involved in adipogenesis (C/EBPβ/α, and SREBP-1c) decreased [66,75]. Furthermore, AMPK, which is increased under CGA influence, stimulates browning by increasing UCP-1 and PGC-1α expression, which are key to the transformation of WAT into BeAT [76]. Another study [77] showed that CGA promotes the differentiation of 3T3-L1 preadipocytes into a brown-like phenotype, and in mature WAT, CGA induces transdifferentiation toward BeAT with higher expression of thermogenic and oxidative genes.

### 2.4. Synergistic Action of CGA with Other Compounds and Weight Loss Effects

Numerous in vivo studies, conducted on both animal models and humans, confirm that CGA—alone or combined with other compounds—positively affects obesity.

In a study conducted on mice with Metabolic Dysfunction-Associated Fatty Liver Disease (MAFLD), it has been shown that the use of Wang’s Metabolic Formula (WMF), which contains chlorogenic acid, geniposide, albiflorin, paeoniflorin, and calycosin-7-O-glucoside, reduces obesity by increasing high-density lipoprotein levels, lowering liver triglyceride levels, and reducing pro-inflammatory cytokines. Western blot analyses have also shown that WMF increases the expression of PPAR-α, PPAR-β, and RXR in the liver [78]. In another study, male mice were given CGA or a dietary supplement containing CGA and chromium (III). After 3 weeks of treatment during a 7-week high-fat diet, both supplements inhibited weight gain [79]. CGA, contained in mulberry leaves inhibited weight gain, reduced fasting blood glucose and Lee’s index in obese mice previously fed a high-fat diet [80,81]. In another study, rats were administered olanzapine—a drug with metabolic disorder and obesity as side effects—followed by CGA or metformin (as a positive control) [82]. Both metformin and CGA were shown to alleviate the effects of the drug, including weight gain, liver damage, elevated fasting glucose, leptin and triglycerides levels [83]. A study focusing on gut microbiota showed that, through its influence on *Akkermansia muciniphila*, CGA inhibits weight gain and dyslipidemia, and enhances the intestinal barrier in mice [84]. The anti-obesity effect of CGA, as one of the components of coffee pulp, was also demonstrated in mice previously fed a high-fat diet [85].

Human studies have also revealed the beneficial effects of CGA on obesity (see Table 1). A dietary supplement called Altilix^®^, which contains chlorogenic acid and luteolin (both of which have anti-obesity effects [86]), was given to participants with pre-obesity in a randomized, double blind study. Altilix^®^ significantly improved lipid profile, metabolic and liver parameters and caused a decrease in waist circumference and body weight. In another double-blind, placebo-controlled clinical study, overweight patients were given chlorogenic acid isomers (CGA-7^TM^) and a significant reduction in body weight and BMI was observed, along with an increase in lean mass and improvement in lipid profile [87].

## 3. Chlorogenic Acid in the Prevention and Management of Hypertension

Chlorogenic acid, as one of the major polyphenols found in coffee exhibits strong antioxidant and anti-inflammatory properties [94]. An increasing body of research highlights its significant role in the prevention and treatment of cardiovascular diseases (CVD), particularly hypertension, through antioxidant activity, improvement of endothelial function, modulation of the renin–angiotensin–aldosterone system (RAAS), platelet activity, and vascular remodeling.

### 3.1. Antioxidant and Anti-Inflammatory Effects

In the context of oxidative stress, CGA has been shown to inhibit NADPH oxidase and reduce the production of ROS, which play a key role in the development of hypertension [95,96,97]. By reducing oxidative stress and inflammation, CGA improves endothelial function and exerts a beneficial effect on blood pressure [98]. Phenolic compounds present in coffee, such as CGA, demonstrate high antioxidant activity, making them potentially effective in hypertension prevention [94].

### 3.2. Endothelial Function and Barrier Integrity

At the endothelial level, CGA increases transendothelial electrical resistance (TEER) and the expression of ZO-1 protein, which is crucial for the integrity of tight junctions [99]. This effect is supported by activation of the Rap1 signaling pathway, which strengthens intercellular junctions, enhances NO production, and reduces inflammation. CGA also promotes NO synthesis by inhibiting arginase and cholinesterase, as well as protecting NO from degradation by free radicals [100].

### 3.3. RAAS Inhibition and Blood Pressure Regulation

An important mechanism of CGA action is inhibition of angiotensin-converting enzyme (ACE), a key component of the RAAS. CGA binds to the active sites of ACE, thereby reducing the production of angiotensin II—a potent vasoconstrictor—which leads to a decrease in blood pressure [100,101]. Evidence from in silico, in vivo, and in vitro studies confirms the efficacy of CGA in inhibiting ACE and limiting the activity of this pathway [102,103]. Furthermore, CGA inhibited medial layer hypertrophy of the aorta and improved endothelial function in spontaneously hypertensive rats [104,105]. These effects were also confirmed in a systematic review and meta-analysis [106].

### 3.4. Molecular Mechanisms in Vascular Protection

At the molecular level, CGA acts through the PI3K/AKT, ERK1/2, and JNK signaling pathways, activates FOXO genes, decreases the expression of pro-inflammatory cytokines and MMPs, and beneficially modulates adiponectin levels as well as PPARα/γ activity [96]. A key role is played by activation of the Nrf2/HO-1 pathway, which contributes to the reduction in oxidative stress, improvement of endothelial function, and protection of blood vessels against damage, including in diabetic models [107,108]. CGA has also been shown to modulate the Nrf2/ARE pathway in response to oxidative stress induced by environmental factors [109]. Literature reviews confirm CGA activity at the levels of Nrf2, NF-κB, and MAPK, along with associated anti-inflammatory effects [14,110].

### 3.5. Antiplatelet and Anti-Thrombotic Activity

The antiplatelet effects of CGA are supported by both in vitro and in vivo studies. Fuentes et al. (2014) demonstrated that CGA, at concentrations of 0.1–1 mmol/L, effectively inhibits platelet aggregation induced by ADP, collagen, and TRAP-6, and reduces the expression of adhesion molecules such as P-selectin and CD40L via activation of adenosine A2A receptors and increased intracellular cAMP levels [111,112]. Similar effects were observed in vivo, where CGA reduced thromboxane A2 levels and inhibited COX-1 activity [111]. Recent nanotechnology-based studies revealed that CGA conjugates may act as effective nano-anticoagulants, also via modulation of the COX-1/TXA2 pathway [113].

### 3.6. Vascular Remodeling and Cardioprotective Effects

CGA also influences vascular remodeling. In studies on isolated rat aortic rings, CGA induced endothelium-dependent relaxation via activation of endothelial nitric oxide synthase (eNOS) and the endothelium-derived hyperpolarizing factor (EDHF) pathway [114]. Moreover, it limited migration and proliferation of vascular smooth muscle cells, counteracting pathological vascular changes [97]. In another review, CGA was recognized as a compound with high cardioprotective potential, considering its antioxidant, anti-inflammatory, and antihypertensive activities [102].

The pharmacokinetics and bioavailability of CGA remain limited; however, new strategies, such as nanocarriers and ethyl ester derivatives, have been shown to improve its distribution to target tissues [107]. This is important from the perspective of CGA’s biological efficacy [115]. In clinical trials, CGA administered as green coffee extract (GCE) at doses of 93–185 mg per day for 4 weeks significantly reduced systolic blood pressure (by 4.7–5.6 mmHg) and diastolic blood pressure (by 3.3–3.9 mmHg) in individuals with mild hypertension (see Table 1) [90,93]. A review of eight clinical trials involving 522 participants showed that this effect occurred mainly in hypertensive individuals, with no clear dose–response relationship [14]. In animal models (spontaneously hypertensive rats), the antihypertensive effect of CGA was dose-dependent and absent in normotensive individuals [105].

From a safety perspective, CGA does not exhibit toxicity with moderate, long-term use. However, attenuation of the antihypertensive effect has been reported with prolonged high-dose administration, suggesting a more favorable effect at lower doses [18,116].

## 4. Chlorogenic Acid and Its Effects on Lipid Metabolism

Chlorogenic acid has attracted considerable attention for its potential role in improving lipid metabolism. Dyslipidemia, characterized by abnormal levels of cholesterol and triglycerides, is a major risk factor for cardiovascular diseases. Recent studies suggest that CGA may influence lipid homeostasis through multiple mechanisms, including enzyme modulation, activation of key metabolic pathways, and anti-inflammatory and antioxidant effects. In this section, we summarize the main molecular pathways by which CGA may contribute to the management of dyslipidemia.

### 4.1. CGA Increases CYP7A1 Enzyme Expression

Cholesterol 7α-hydroxylase (CYP7A1) is a hepatic enzyme essential for bile acid synthesis, the primary pathway for cholesterol catabolism. Li et al. (2011) demonstrated that mice with elevated CYP7A1 expression showed increased bile acid production and secretion compared with wild-type mice [117]. In another study, overexpression of CYP7A1 in mice was associated with reduced LDL cholesterol levels [118]. Ye et al. further reported that mice fed a CGA-rich diet showed significantly increased CYP7A1 expression [119]. These findings suggest that CGA may contribute to LDL cholesterol reduction in individuals with metabolic syndrome.

### 4.2. CGA Activates AMPK

AMPK is an enzyme involved in prompting a cell to respond to a decreased level of available energy [120]. Apart from AMP, which is its default activator [121] and ADP, which also indicates that the cell is experiencing energy stress [122], various other substances can cause AMPK activation [123]. Some substances can activate AMPK directly by acting as AMP analogs. These include the naturally occurring salicylate [124] as well as synthetic compounds such as A-769662 [125], 14d [126] or 5-aminoimidazole-4-carboxamide ribonucleoside [127]. Apart from direct allosteric AMPK activators, various substances can activate (or inactivate) AMPK indirectly, by shifting the balance between ATP, ADP and AMP [123]. These include synthetic pharmaceuticals such as metformin, phenformin or phenobarbital, as well as naturally occurring substances such as resveratrol, berberine [128], and, discussed in this article, CGA [129]. In vitro, CGA causes the activation of AMPK in the calmodulin-dependent protein kinase kinase-β (CaMKKβ) pathway [130]. Furthermore, CGA can lower the expression of CD36 on certain cells, which leads to AMPK activation as well [131]. Once activated, AMPK exerts a pleiotropic effect upon the cell, leading to inhibition of energy-consuming anabolic processes with simultaneous activation of ATP-producing catabolic pathways [120]. The effects of AMPK activation responsible for increasing lipid catabolism include decreasing the activity of SREBP [132], and, in turn, acetyl-CoA carboxylase (ACC) enzymes [133,134], described in more detail below, as well as other enzymes involved in lipid metabolism, such as GPAT [121], HSL [135] and HMGCR [136].

### 4.3. CGA Modifies the Expression of Lipid Metabolism Enzymes

Fatty acid synthase (FAS) is the key enzyme in the elongation of saturated fatty acid chains, while (ACC) provides substrates for this process [137]. The expression of these enzymes is mainly regulated by PPARs. Activation of PPAR-γ stimulates lipogenesis by increasing FAS and ACC activity [5]. On the other hand, PPAR-α activation promotes lipid catabolism [138]. A recent zebrafish study showed that a CGA-rich diet downregulated PPAR-γ, ACC, and FAS, while upregulating PPAR-α activity [139]. Given the similarities in lipid metabolism between zebrafish and humans, these results indicate potential benefits of CGA in dyslipidemia treatment, although further human studies are necessary.

### 4.4. CGA Influences the Lipid Profile via Antioxidant and Anti-Inflammatory Action

Apart from shifting the expression of enzymes responsible for lipid metabolism, CGA may also be capable of maintaining lipid homeostasis through its antioxidant and anti-inflammatory properties [6]. Numerous studies have described CGA’s antioxidant and anti-inflammatory capabilities both in vitro [140,141,142,143] and in vivo in rat [140] and mouse [143] models. The effect of CGA supplementation on lipid profile has been assessed in human studies, though sometimes with conflicting results (see Table 1). However, no studies on humans assess whether it is the antioxidant and anti-inflammatory properties of CGA that are responsible for the changes it may cause in lipid fraction concentrations. Nevertheless, the role of these properties in influencing the lipid profile is believed to be substantial [144], though further studies are necessary to confirm this hypothesis.

## 5. Chlorogenic Acid as a Modulator of Diabetic Pathophysiology

**Figure 3:**
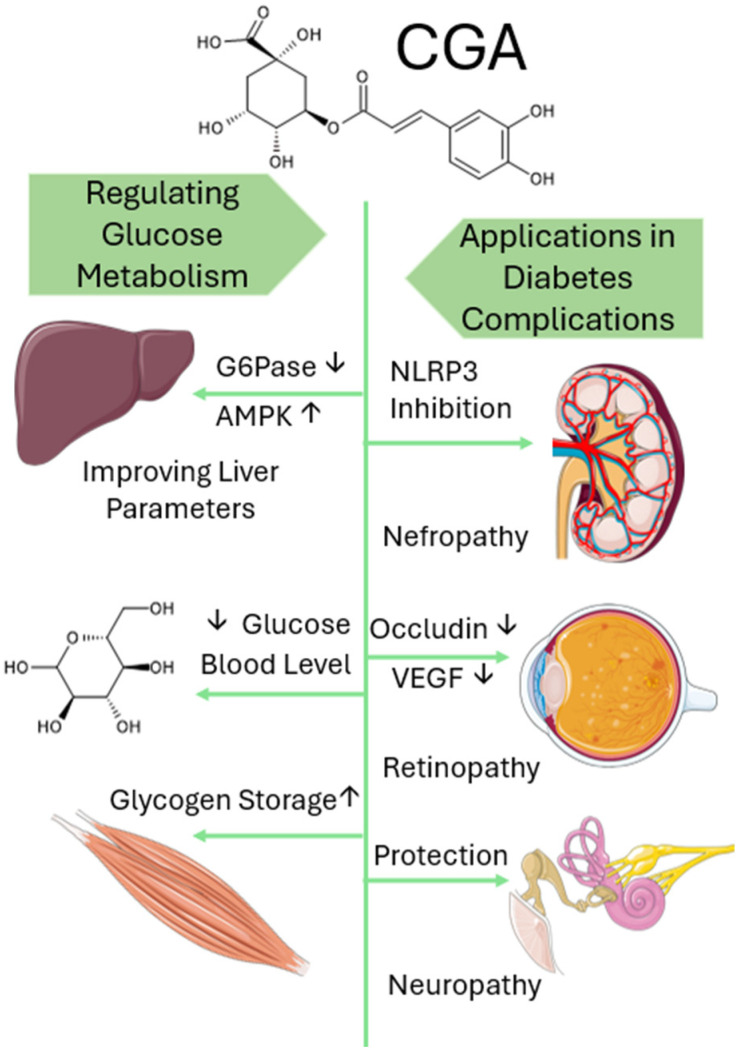
Mechanism of Chlorogenic Acid Action in Reducing Glucose Levels and Preventing Diabetes Complications. CGA lowers blood glucose via insulin signaling, AMPK activation, and reduced glucose absorption, while protecting against diabetes complications through antioxidant, anti-inflammatory, and tissue-protective effects. Upward arrows (↑) indicate an increase or activation of the parameter, while downward arrows (↓) indicate a decrease or inhibition.

Chlorogenic acid, widely present in plant-based foods, is also an important constituent of traditional Chinese medicine preparations, and in recent years, it has been shown to possess hypoglycemic, hypolipidemic, anti-inflammatory, antioxidant, and other pharmacological properties [145]. In particular, CGA alleviates the effects of type 2 diabetes mellitus (DM) and helps prevent its development, while also exerting anti-obesity effects, modulating gut microbiota, and improving lipid profiles, thereby contributing to systemic metabolic balance. Moreover, CGA also exhibits beneficial effects against diabetes-related complications such as diabetic nephropathy, diabetic retinopathy, and diabetic peripheral neuropathy (see Figure 3) [146,147]. This chapter reviews the use of CGA in the prevention and treatment of type 2 diabetes and its complications, thereby providing a foundation for further research and medical applications [14,148].

### 5.1. Chlorogenic Acid’s Role in Regulating Glucose Metabolism

Chronic hyperglycemia is a defining feature of DM. In the early stages of the disease, pancreatic β-cells are overworked in an attempt to regulate glucose levels, leading to cellular stress and eventual dysfunction [149]. This prolonged hyperglycemic state contributes significantly to glucose toxicity and the development of long-term diabetic complications. CGA has shown promise in helping manage blood glucose levels [150].

#### 5.1.1. Clinical Studies (Human Trials)

In a controlled clinical trial, individuals with impaired glucose tolerance who received 400 mg of CGA three times daily over 12 weeks experienced noticeable reductions in fasting glucose levels [89]. Similarly, in another clinical study, participants receiving green coffee bean extract (rich in CGA, 400 mg capsules twice daily for eight weeks) exhibited improved metabolic profiles [119]. In one clinical study, CGA supplementation for 12 weeks led to reductions in both fasting blood glucose and insulin secretion, indicating improved insulin sensitivity and reduced resistance [14]. However, another trial found that CGA did not significantly enhance the secretion of glucagon-like peptide-1 (GLP-1) or glucose-dependent insulinotropic peptide (GIP) [151].

#### 5.1.2. In Vivo Studies (Animal Models)

Multiple in vivo studies support these findings. Mice on high-fat diets exhibited significantly reduced glucose levels after six weeks of supplementation with CGA-rich green coffee extract (100 mg/kg body weight) [119]. In type 2 diabetic mice, Simiao Wan (SMW) and its bioactive ingredients (including CGA and berberine) reduced fasting glucose and lipid levels, improved insulin levels, and activated the IRS1/AKT2/GLUT2 signaling pathway while inhibiting FOXO1, confirming the glucose-lowering effects of CGA through enhanced hepatic insulin signaling [152]. Rats with type 2 diabetes treated with mulberry leaf extract (containing CGA), rutin, or isoquercitrin showed that the extract and pure CGA effectively lowered blood glucose, whereas isoquercitrin had minimal effect, indicating CGA and rutin as the active compounds [152]. In db/db mice, CGA exhibited effects comparable to metformin, improving glucose tolerance and reducing hepatic lipogenesis via upregulation of β-oxidation-related genes (CPT1a, ACOX1) and antioxidant enzymes (SOD1, SOD2, GPX1), as well as modifying gut microbiota composition [6]. Further, 12-week administration of CGA (80 mg/kg/day) improved glucose control and increased glycogen stores in muscle tissue [130,151]. In another study, low-dose CGA (5 mg/kg/day for 45 days) resulted in blood glucose levels nearly three times lower than those in the untreated diabetic control group [152,153].

#### 5.1.3. In Vitro Studies (Cellular Mechanisms)

CGA enhanced insulin signaling in β-cells by activating CREB-dependent IRS-2 expression, reducing endoplasmic reticulum (ER) stress, and protecting β-cell function and mass [150]. Mechanistically, CGA regulates glucose metabolism by inhibiting glucose-6-phosphatase (G6Pase) in the liver, delaying intestinal glucose absorption through inhibition of α-glucosidase, and enhancing peripheral glucose uptake via activation of AMPK, a key regulator of cellular energy balance. Additionally, in silico studies demonstrated CGA binding affinities to PPAR-γ and α-amylase, suggesting improved insulin sensitivity and reduced postprandial glucose spikes [143].

### 5.2. Application in Diabetes Complications

CGA not only modulates glucose metabolism but also provides protection against complications of diabetes, such as diabetic nephropathy, retinopathy, neuropathy, muscle atrophy, and vascular damage [154,155,156].

Diabetic nephropathy (DN) is one of the most common microvascular complications of diabetes [157]. There have been efforts to explore the use of CGA in the prevention and treatment of DN. A study [147] shows that CGA, by increasing nuclear Nrf2 translocation, promotes antioxidant enzymes expression. Studies on diabetic rats have shown that CGA reduces malondialdehyde (MDA) levels in the kidneys, increases the activity of antioxidant enzymes such as superoxide dismutase (SOD) and glutathione peroxidase (GSH-Px), and decreases the expression of factors involved in oxidative stress and inflammatory responses in the kidney, including IL-6, TNF-α, and IL-1β [158]. In vitro and in vivo studies further revealed that CGA inhibits Notch1 and Stat3 signaling, reducing lipid accumulation and renal fibrosis, downregulating SREBP1c, and upregulating CPT1A, thereby enhancing fatty acid oxidation [159]. Histopathological analysis revealed that CGA reduces glomerular hypertrophy and mesangial matrix expansion. Another study confirmed these findings, demonstrating that CGA enhances the activity of SOD, GSH-Px, and catalase (CAT) in the kidneys, lowers MDA levels, downregulates the expression of (COX-2), and inhibits the proliferation and expansion of mesangial cells [160]. These results suggest that chlorogenic acid may help prevent and treat diabetic nephropathy by alleviating oxidative stress and inflammatory responses in the kidneys. CGA also inhibits the NLRP3 inflammasome via Nrf2 activation, significantly decreasing proteinuria, creatinine, and urea levels in diabetic rats—an effect abolished upon Nrf2 gene silencing [161].

Diabetic retinopathy (DR) is a microvascular complication of diabetes and is a leading cause of vision impairment in middle-aged and elderly individuals worldwide [162,163]. Treatment of diabetic mice with honeysuckle extract, which high-performance liquid chromatography has shown to primarily contain (CGA), suppressed streptozotocin (STZ)-induced retinal vascular proliferation and reduced serum levels of (VEGF) [164]. Furthermore, in both cellular and animal studies, CGA counteracted the effects of hypoxia-inducible factor 1-alpha (HIF-1α) and decreased VEGF expression during DR, leading to improved abnormal retinal neovascularization. These findings were supported by retinal immunofluorescence staining of differentiation clusters and histopathological observations [165]. Additionally, in diabetic rats, CGA restored reduced levels of occludin, a tight junction protein essential for the blood-retinal barrier, and inhibited VEGF expression [166]. In summary, these results suggest that chlorogenic acid may alleviate the effects of diabetic retinopathy, particularly by improving retinal vascular permeability.

Furthermore, in diabetic mice, CGA improved the auditory function of the external auditory canal, alleviated dysfunction in the central auditory pathway, contributed to the regeneration of damaged outer hair cells in the cochlea, prevented neuroma formation, and protected the ear hair cells [167]. CGA also protected against auditory neuropathy and neuroma formation [168].

Moreover, in the mechanical claw pressure test, CGA effectively relieved diabetic neuropathic pain, likely by lowering blood glucose levels and reducing oxidative stress [169].

### 5.3. Muscle Atrophy and Tissue Protection

CGA supplementation (12.5 mg/kg) in diabetic rats significantly upregulated expression of antioxidant and metabolic genes (SOD-1, SOD-2, PGC-1α, calcineurin) and improved muscle histology, indicating a protective role in diabetes-induced muscle atrophy [170].

### 5.4. Wound Healing and Skin Complications

Innovative therapeutic applications include electrospun nanofiber wound dressings with CGA, which showed both hypoglycemic and regenerative effects in diabetic foot ulcer models [171].

## 6. Bioavailability and Stability of Chlorogenic Acid

Chlorogenic acid exhibits limited oral bioavailability, a factor that substantially constrains its translational and therapeutic potential. This limitation arises primarily from its hydrophilic character, chemical instability, and extensive presystemic metabolism in the gut and liver. CGA is prone to hydrolysis—particularly under alkaline conditions—yielding caffeic and quinic acids, and although it remains relatively stable in the acidic gastric environment, it undergoes partial degradation and limited trans-epithelial absorption in the small intestine [16,172,173]. Pharmacokinetic studies in humans have consistently shown low systemic exposure after oral ingestion. Following consumption of coffee or green coffee extract, reported maximum plasma concentrations (Cmax) typically remain below 1–5 µmol/L, with an elimination half-life (t½) of approximately 0.5–1.5 h, indicating rapid metabolism and clearance. Orally administered CGA undergoes hydrolysis by intestinal and hepatic esterases, generating caffeic acid that is further subjected to methylation, glucuronidation, and sulfation before renal excretion. As a result, most circulating species are phase II metabolites rather than the parent compound [172,174]. The gut microbiota also plays a key role in determining CGA’s metabolic fate. Bacteria of the genera Eubacterium, Bifidobacterium, and Clostridium catalyze the conversion of CGA into various phenolic metabolites, such as ferulic, dihydroferulic, and 3-hydroxyphenylacetic acids. These microbial metabolites can display different levels of bioactivity and bioavailability compared to the parent compound, and in some cases, they may mediate part of CGA’s antioxidant, vasoprotective, or anti-inflammatory effects observed in vivo [175,176,177]. Consequently, interindividual variability in microbiome composition significantly affects both the pharmacokinetics and pharmacodynamics of CGA. From a physicochemical perspective, CGA is water-soluble but highly unstable when exposed to elevated temperature, light, oxygen, or alkaline pH. These factors lead to oxidation and degradation, reducing the intact fraction available for absorption [178]. To overcome this instability and improve systemic delivery, several formulation strategies have been explored. Lipid-based nanocarriers (such as nanostructured lipid carriers and liposomes) and polymeric nanoparticles can protect CGA from degradation and provide controlled intestinal release. Complexation with cyclodextrins (β-CD, HP-β-CD) enhances solubility and chemical stability [179], whereas esterification into more lipophilic derivatives increases membrane permeability and resistance to enzymatic hydrolysis. Additional strategies include co-formulation with enzyme inhibitors to reduce intestinal metabolism or the design of enteric-coated and gastro-retentive dosage forms to optimize absorption sites [178]. Moreover, non-oral delivery routes have been examined to bypass presystemic metabolism. In early phase I clinical studies, intravenous formulations of CGA have demonstrated improved systemic exposure and pharmacokinetic profiles, although their safety and therapeutic efficacy require further validation. In summary, the limited oral bioavailability of CGA (Cmax < 5 µmol/L, t½ ≈ 1 h) results from its chemical instability, enzymatic hydrolysis, and extensive conjugative and microbiota-dependent metabolism [16,177,180]. Nevertheless, advances in nanocarrier systems, cyclodextrin complexation, and ester derivatization represent promising approaches to enhance CGA stability, absorption, and therapeutic applicability.

Chlorogenic acid generally exerts beneficial effects on the organism when consumed at typical dietary levels. However, acute or subacute exposure to high doses may induce toxic effects. In an experimental study in mice, administration of CGA at doses ranging from 30 to 1000 mg/kg resulted in elevated oxidative stress markers, impaired liver function, and histopathological alterations, with these effects being particularly pronounced at approximately 240 mg/kg [181]. Similarly, a randomized crossover clinical study reported that oral administration of 2 g of CGA per day for 7 days led to an increase in total homocysteine levels (approximately +12% post-administration), indicating potential clinical implications at very high doses [18]. Overall, based on available data from toxicological, pharmacokinetic, and clinical studies, no adverse effects have been observed at doses corresponding to normal dietary intake, and acute or long-term exposure to standard amounts of CGA does not appear to pose a health risk [182]. In vitro studies have further demonstrated that CGA, frequently in combination with caffeic acid, can enhance the expression of browning-associated markers (UCP1, PGC-1α, CD137), activate the AMPK signaling pathway, and modulate PPARγ in human SGBS adipocytes; however, to date, these effects have not been confirmed in vivo in humans [101].

## 7. Conclusions

Chlorogenic acid is an important polyphenol present in the human diet, playing a crucial role in maintaining metabolic balance and preventing lifestyle-related diseases. It exhibits pleiotropic activity, including antioxidant, anti-inflammatory, hypoglycemic, hypolipidemic, and cardioprotective effects. The mechanisms of its activity include, among others, the reduction in oxidative stress through activation of the Nrf2 pathway and inhibition of NF-κB, regulation of energy processes and lipid metabolism via AMPK activation, as well as modulation of adipogenesis, lipolysis, browning of adipose tissue, and thermogenesis. In addition, CGA positively influences the gut microbiota by increasing the abundance of probiotic strains and stimulating the production of short-chain fatty acids, which contributes to improved intestinal barrier function, reduced endotoxemia, and mitigation of chronic inflammation.

**Figure 4:**
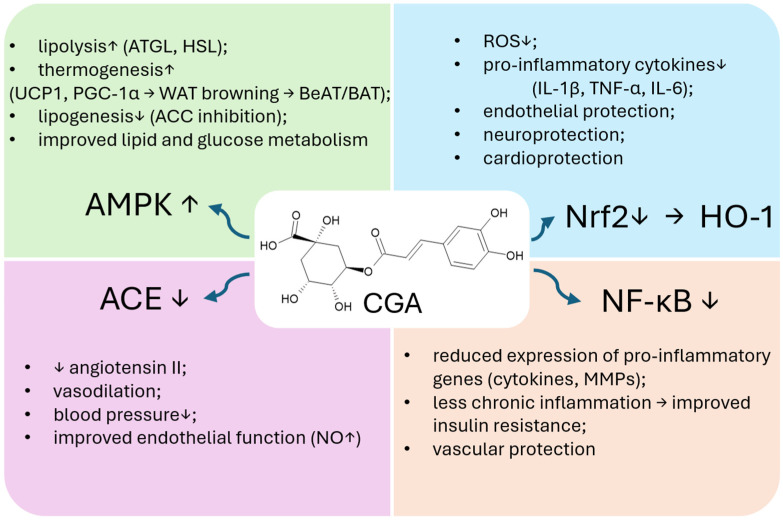
Integrated diagram of CGA-mediated signaling pathways (AMPK, Nrf2/HO-1, NF-κB, RAAS) and their metabolic outcomes. Upward arrows (↑) indicate an increase or activation of the parameter, while downward arrows (↓) indicate a decrease or inhibition.

These beneficial effects are mediated through interconnected molecular pathways that underlie CGA’s pleiotropic actions (see Figure 4). Activation of the AMPK signaling pathway enhances mitochondrial biogenesis, stimulates fatty acid oxidation, and suppresses lipogenesis and adipocyte differentiation, thereby improving energy expenditure and insulin sensitivity. Concurrently, activation of the Nrf2/HO-1 pathway and inhibition of NF-κB reduce oxidative stress and chronic inflammation, protecting vascular and metabolic tissues. CGA also modulates PPARα/γ, PI3K/Akt, and MAPK cascades, which integrate antioxidant, anti-inflammatory, and metabolic responses, leading to improved lipid homeostasis, endothelial function, and regulation of the renin–angiotensin–aldosterone system RAAS. Together, these signaling pathways form a cohesive mechanistic framework explaining CGA’s multifaceted effects in metabolic syndrome.

In the context of metabolic disorders, numerous preclinical and clinical studies indicate that CGA improves insulin sensitivity, regulates blood glucose levels, lowers blood pressure, and positively affects the lipid profile by reducing LDL cholesterol and triglyceride levels while increasing HDL cholesterol [87,88,89,90,91,92,93,183]. In overweight and obese individuals, CGA supplementation has been shown to promote reductions in body weight and waist circumference, as well as improve liver function parameters. An important aspect of its action also concerns the cardiovascular system—CGA enhances endothelial function, inhibits angiotensin-converting enzyme activity, supports nitric oxide synthesis, and exhibits antiplatelet properties, making it a potential adjuvant in the treatment of hypertension and atherosclerosis. Moreover, CGA shows beneficial effects in the prevention and progression of diabetic complications such as nephropathy, retinopathy, and neuropathy, where its antioxidant and anti-inflammatory actions help limit tissue and vascular damage.

A distinctive feature of CGA’s action, compared with many other polyphenols, is its ability to simultaneously modulate the Nrf2/HO-1 axis and pro-inflammatory pathways (including NF-κB and NLRP3), resulting in tissue-specific protective effects. In diabetic models, CGA has been shown to increase the activity of antioxidant enzymes—such as SOD, GSH-Px, and catalase—and to reduce the levels of lipid peroxidation products in the kidneys, which correlates with improved nephrological parameters. At the same time, NLRP3 inhibition through Nrf2 activation is associated with reduced proteinuria and improved kidney function. In the retina, CGA decreases VEGF expression and restores the levels of tight junction proteins, thereby limiting excessive vascular permeability. Additionally, intestinal metabolites of CGA and microbiota alterations contribute to these effects by enhancing the antioxidant system and reducing endotoxemia. Thus, the therapeutic efficacy of CGA results from the synergy of antioxidant enzyme induction, NLRP3 inflammasome suppression, and inflammatory cytokine modulation.

Despite the numerous documented health benefits of CGA, its broader application is limited by relatively low bioavailability, resulting from gastrointestinal metabolism and dependence on gut microbiota. Therefore, one of the key directions of future research is the development of novel pharmaceutical and nutraceutical formulations (e.g., nanocarriers, esters, cyclodextrin complexes) to improve the stability and bioavailability of this compound. Another important area of research is the analysis of the synergistic effects of CGA with other plant-derived bioactive compounds, which may enhance its metabolic and protective actions.

In summary, chlorogenic acid has significant potential as a component of functional foods and dietary supplements, as well as an adjunct compound supporting conventional therapy of chronic diseases associated with metabolic dysfunction. Its safety, confirmed in both animal and clinical studies, increases its attractiveness for long-term use in prevention and treatment. However, broader clinical trials will be necessary in the future to better determine the optimal doses, duration of use, and effectiveness of CGA across different patient populations. Thus, CGA may become one of the key natural compounds supporting personalized medicine in the field of metabolic and cardiovascular diseases.

## Figures and Tables

**Table 1:** Comparison of the influence of CGA on selected parameters depending on the dose and duration of treatment. The “Results” section depicts changes within study groups over the course of CGA treatment, expressed in percent change. The data of little statistical value (due to a *p*-value > 0.05, uncertainties regarding parameter calculation or similar changes being observed within the control group over the course of the study) were marked with a lighter color than the rest. n, sample size; BM, body mass; BMI, body mass index; WC; waist circumference; FBG, fasting blood glucose; A1c, glycated hemoglobin HbA1c; TC, total cholesterol; HDL, high density lipoprotein; LDL, low density lipoprotein; SBP, systolic blood pressure; DBP, diastolic blood pressure; HHQ, hydroxyhydroquinone. x—Parameter not assessed in the study; *—Mild hypertension defined as SBP 140–159 and DBP 90–99; **—Mild hypertension defined as SBP 140–155 and DBP 90–97.

Patient Data		Intervention Details				Results											Ref. Number
Primary Illness	n	Dosage (Pure CGA)	CGA Origin	Form	Treatment Duration	BM [%]	BMI [%]	WC [%]	FBG [%]	A1c [%]	TC [%]	HDL [%]	LDL [%]	TG [%]	SBP [%]	DBP [%]	
Pre-obesity (BMI 25–30)	28	15–18 mg × 1/day	Nutraceutical (CGA + luteolin)	tablet	6 months	−2.63	−3.01	−3.15	x	−0.73	−12.60	2.09	−15.40	−18.40	x	x	[88]
Pre-obesity (BMI 25–30)	33	250 mg × 2/day	GCE (decaf.)	capsule	12 weeks	−2.74	−2.91	−1.03	−0.68	−1.84	−4.84	4.55	−6.25	−4.92	x	x	[87]
Impaired glucose tolerance	14	400 mg × 3/day	GCE	capsule	12 weeks	−3.00	−3.68	−1.92	−3.50	0	−4.44	58.82	−17.39	−18.75	−3.90	−1.74	[89]
Mild hypertension (SBP 140–159, DBP < 99)	9	300 mg × 1/day	HHQ-reduced coffee	adjusted coffee	8 weeks	−0.73	−0.83	x	−3.83	1.89	3.87	3.75	−2.26	26.75	−6.25	0.90	[90]
Mild hypertension *	29	24.84 mg × 1/day	GCE	enriched soup	28 days	−0.14	−0.40	x	x	x	2.10	0	2.48	1	−2.19	−3.15	[91]
Mild hypertension *	28	50.22 mg × 1/day	GCE	enriched soup	28 days	−0.14	0	x	x	x	−3.44	0	−3.82	1.78	−3.22	−3.46	[91]
Mild hypertension *	31	99.9 mg × 1/day	GCE	enriched soup	28 days	0	0	x	x	x	−3.49	0	−6.50	−3.93	−3.84	−4.22	[91]
Mild hypertension *	14	140 mg × 1/day	GCE	enriched fruit juice	12 weeks	x	0	x	8.99	x	0	7.27	5.55	−5	−6.90	−7.69	[92]
Mild hypertension **	41	82 mg × 1/day	HHQ-depleted coffee	adjusted coffee	4 weeks	x	x	x	x	x	x	x	x	x	−1.86	−2.96	[93]
Mild hypertension **	40	172 mg × 1/day	HHQ-depleted coffee	adjusted coffee	4 weeks	x	x	x	x	x	x	x	x	x	−1.96	−2.53	[93]
Mild hypertension **	40	299 mg × 1/day	HHQ-depleted coffee	adjusted coffee	4 weeks	x	x	x	x	x	X	x	x	x	−2.29	−3.07	[93]

## Data Availability

The original contributions presented in this study are included in the article. Further inquiries can be directed to the corresponding author.
